# Large Solvent‐Dependent Rate Enhancement of Autocatalytic Hydrolysis of Alkyl and Aryl Thiosulfates

**DOI:** 10.1002/open.202500552

**Published:** 2026-04-09

**Authors:** Fatema B. Amin, Katherine M. Kressler, Gregory S. Ferguson

**Affiliations:** ^1^ Department of Chemistry Lehigh University Bethlehem Pennsylvania USA; ^2^ Department of Materials Science & Engineering Lehigh University Bethlehem Pennsylvania USA

**Keywords:** autocatalysis, hydrolysis, organic thiosulfates, rate enhancement

## Abstract

The hydrolysis of alkyl and aryl thiosulfates (Bunte salts), to produce the corresponding thiol and bisulfate ion, is autocatalytic in the absence of added acid and proceeds at greatly accelerated rates in organic solvents at room temperature relative to the same reaction in aqueous solution. Kinetics studies of the hydrolysis of *n*‐hexadecyl thiosulfate and *p*‐nitrophenyl thiosulfate allowed the determination of the associated rate constants. Control experiments indicate that the enhancement of the rate was not due to the preconcentration of the reactant and the bisulfate catalyst within reverse micelles. Rather, it appears to be related to the properties of the solvent and differences in solvation of the reactant, product, and/or transition state. High‐throughput measurements of the induction periods across a reaction population were fit well by a Gaussian function.

## Graphical abstract

Solvent‐dependent rate enhancement of hydrolysis: Hydrolysis of organic thiosulfates is very slow in water, even in the presence of an acid catalyst. Its rate is accelerated by orders of magnitude, however, in THF containing only a trace of water and no acid, where it displays autocatalytic kinetics. This dramatic rate enhancement appears to depend on differences in solvation, not the formation of reverse micelles.

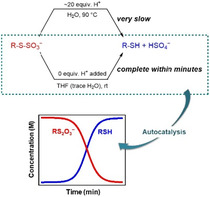

## 1 Introduction

Few, if any, reactions are more fundamental to a broad range of subdisciplines in chemistry and biochemistry than hydrolysis, and understanding them is commensurately important. While the hydrolysis of amphiphilic compounds is frequently reported, kinetic studies have often been confined to acid‐ or base‐catalyzed reactions under harsh conditions and with low reaction rates. During our studies of alkyl thiosulfates (RSSO_3_M, Bunte salts) [1] as precursors for the regioselective, electrochemical formation of thiolate monolayers on gold [2, 3, 4, 5, 6], we noted the spontaneous, nonelectrochemical formation of self‐assembled monolayers (SAMs) on gold derived from these compounds in THF solutions that did not contain the supporting electrolyte, NBu_4_BF_4_ [7]. A previous paper by Lukkari et al. had reported similar results in other solvents [8]. Mechanistic investigations revealed that the hydrolysis of the alkyl thiosulfate by trace water to produce the corresponding surface‐active thiol is actually responsible for the formation of SAMs in this system (Equation (1)) [7, 9, 10]. The presence of BF_4_^−^ inhibited the hydrolysis, presumably by

(1) $${\mathrm{RS}}_{2}{O_{3}}^{‐}+H_{2}O\;\to \;\mathrm{RSH}+{{\mathrm{HSO}}_{4}}^{‐}$$

sequestering water as hydronium ions [11, 12]. The present study focuses on the spontaneous solution‐phase behavior of these salts and the surprisingly large rate enhancement of their hydrolysis to produce thiols.

**SCHEME 1:**
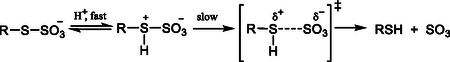
Kice et al. proposed mechanism for the acid‐catalyzed hydrolysis of aliphatic and aromatic thiosulfates to produce the corresponding thiols in 60% dioxane‐water.

The acid‐catalyzed hydrolysis of alkyl thiosulfates in water was first reported by Kice et al., who investigated the kinetics of hydrolysis for the ethyl derivative in aqueous acid [13]. They reported the reaction to be first order in both the substrate and acid, with a pseudo‐first‐order rate constant of 5.7 × 10^−5^ s^−1^ at 96°C in the presence of 1 M strong acid, corresponding to a half‐life of over 3 h under these conditions. The activation parameters (Δ*H* ^‡^  = 31 ± 0.7 kcal/mol and Δ*S* ^‡^  = 5.8 ± 2.0 e.u.) suggest a room‐temperature rate constant of 4.2 × 10^−9^ s^−1^ and thus a half‐life of 5.2 years! In 60% dioxane‐water as the solvent, the rate constant increased to 2.82 × 10^−4^ s^−1^ [14], or a half‐life of 41 min at a lower temperature (69.7°C) and somewhat reduced acid concentration (0.81 M). The authors attributed this rate enhancement in the hydrolysis reaction to the difference in activation entropy in the two solvents. In the same solvent mixture, the aromatic thiosulfate, pyridinium p‐nitrophenyl thiosulfate, showed a pseudo‐first order rate constant of 8.10 × 10^−5^ s^−1^, or a half‐life of over 2 h, for hydrolysis with 0.81 M strong acid at 69.7°C. Their proposed mechanism in dioxane‐water is shown in Scheme 1.

Given the slow kinetics of these reactions at room temperature in the presence of large amounts of both water and acid, we were struck by the speed of the reaction in THF containing only a trace amount of water and no added acid. In this case, the bisulfate in Equation (1) would be provided by the reaction of SO_3_ with water. In the present study, the initially uncatalyzed hydrolysis of alkyl and aryl thiosulfates in THF without added water or acid reached completion within minutes at room temperature. A similar solvent‐dependent rate enhancement has been reported for the hydrolysis of anionic phosphate monoesters and diesters in other organic solvents such as cyclohexane [15], acetone [16], alcohols [17], acetonitrile [18], and dimethyl sulfoxide [19, 20] containing varying concentrations of water, relative to that in water alone. Stockbridge and Hengge attributed the dramatic rate enhancement to a more favorable enthalpy of activation, resulting from desolvation‐induced destabilization of the reactant when hydrolysis occurs in a less polar solvent than water [16, 19, 21].

## 2 Results and Discussion

**FIGURE 1:**
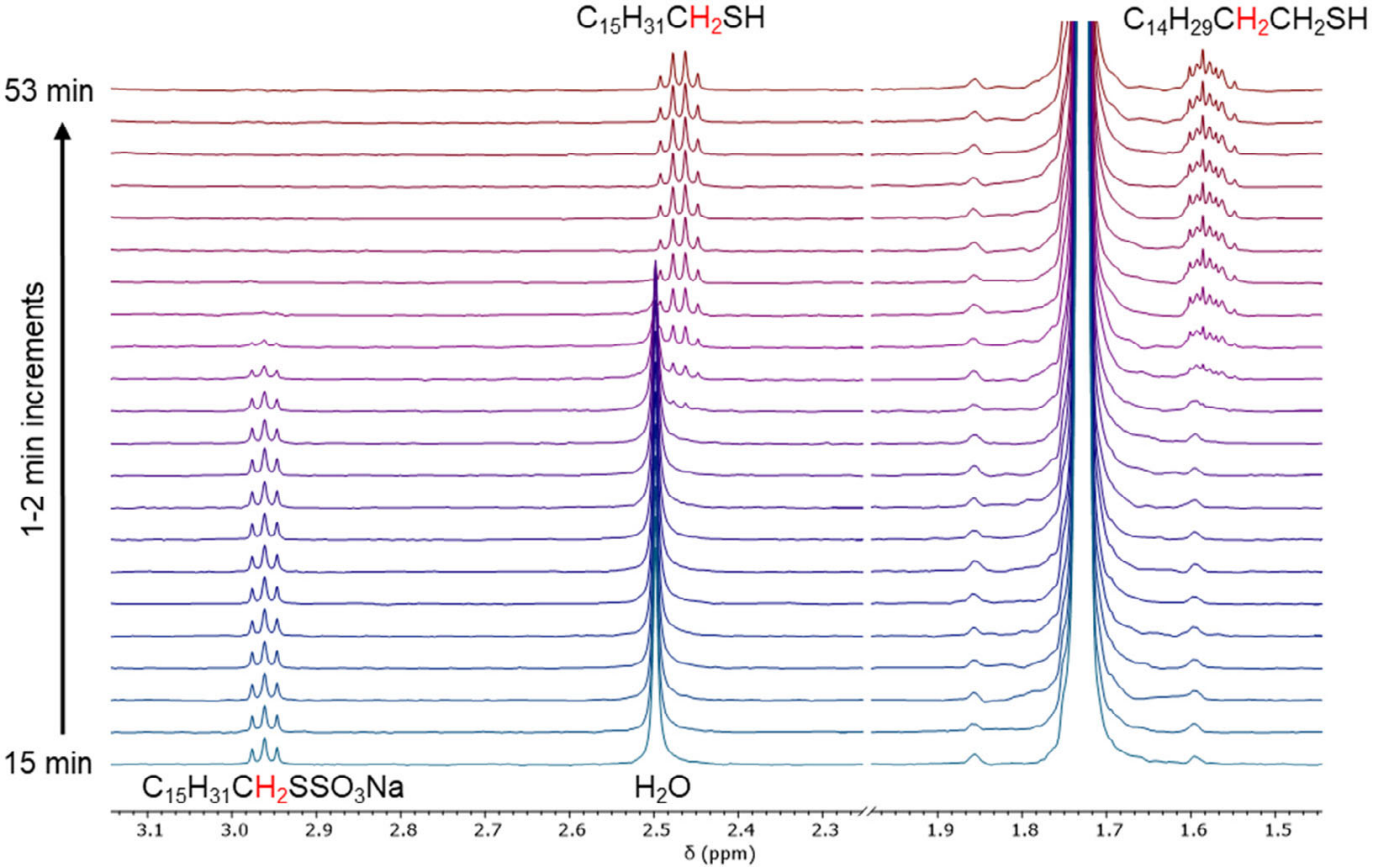
Time‐resolved ^1^H‐NMR scans of 4.67‐mM **1a** in THF‐d_8_ at 1–2‐min intervals. The resonance due to the *β*‐CH_2_ of the thiosulfate is obscured by the residual protons of the solvent at  ∼1.70–1.76 ppm.

In the absence of added acid, the bisulfate byproduct renders the reaction in Equation (1) autocatalytic. As a result, the kinetics of the hydrolysis of sodium hexadecyl thiosulfate (**1a**) in THF showed a sigmoidal profile with a pronounced induction period, followed by a rapid conversion to the product hexadecanethiol (**2a**). We interpret the induction period as reflecting a slow rate of reaction until a sufficient concentration of acid accumulates to catalyze the subsequent exponential product‐time phase of the reaction. Time‐resolved ^1^H NMR spectra of a 4.67 mM solution of thiosulfate **1a** in THF‐d_8_ (≥99.5%) at 1–2‐min intervals at 25°C are shown in Figure 1, with the onset of hydrolysis at between 32 and 36 min (Figure 1). During the reaction, the disappearance of the resonances due to the *α*‐CH_2_ protons adjacent to the thiosulfate group at 2.96 ppm coincides with the growth of resonances due to the *α*‐CH_2_ protons of the thiol at 2.47 ppm and the *β*‐CH_2_ protons of the thiol at just below 1.60 ppm. The hydrolysis reaction and concomitant acidification of the solution also led to attenuation, and then loss, of the water signal at ∼ 2.49 ppm in the NMR spectra in Figure 1. The water present (3.9 equivalents) was due to that in the solvent as supplied by the supplier and that crystallized with the reactant thiosulfate (Figure S4). The water of crystallization accounted for 0.79 equivalents, as determined by the thermogravimetric analysis in Figure S3.

**FIGURE 2:**
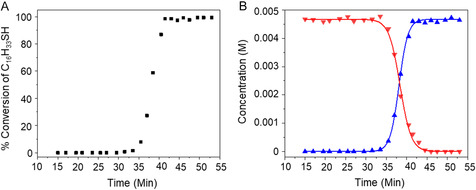
(A) Percent conversion for the hydrolysis of 4.67‐mM hexadecyl thiosulfate, **1a**, in THF to produce hexadecanethiol, **2a**. (B) Concentration profiles of hexadecyl thiosulfate **1a** (red) and hexadecanethiol **2a** (blue) during the reaction. The lines are fits to Equations (3) and (2).

Using the integration of the unobstructed *α*‐CH_2_ resonances for thiosulfate **1a** in the NMR spectra, its concentration was calculated to monitor the course of the reaction. The *α*‐CH_2_ resonances of product **2a** partially overlapped the H_2_O peak until the latter disappeared, so for that portion of the kinetics, the concentration of **2a** was calculated from the change in concentration of **1a**. After that point, the unobstructed *α*‐CH_2_ resonances for **2a** were used. Figure 2A shows the percent conversion of thiosulfate to thiol as a function of time, with a sigmoidal kinetics curve characteristic of autocatalysis, and Figure 2B provides the corresponding concentration profiles of reactant and product. The thiol *α*‐CH_2_ protons remained undetectable during the initial induction period phase, followed by the onset of hydrolysis and rapid conversion. The product concentration for a second‐order autocatalytic reaction, in which acid produced by the reaction catalyzes the formation of the product, can be mathematically expressed in terms of the observed rate constant (*k*), reactant and product concentrations, and time (*t*), as shown in Equation (2) [22]. An expression for the corresponding reactant concentration is given in Equation (3). Concentrations calculated

(2) $$[R–\mathrm{SH}]=\frac{{\left[{\text{NaHSO}}_{4}\right]}_{0}+{\left[{\mathrm{RS}}_{2}O_{3}\mathrm{Na}\right]}_{0}}{1+\frac{{\left[{\mathrm{RS}}_{2}O_{3}\mathrm{Na}\right]}_{0}}{{\left[{\text{NaHSO}}_{4}\right]}_{0}}\exp\;(-kt({\left[{\text{NaHSO}}_{4}\right]}_{0}+{\left[{\mathrm{RS}}_{2}O_{3}\mathrm{Na}\right]}_{0}))}$$

(3) $$[{\mathrm{RS}}_{2}O_{3}\mathrm{Na}]=\frac{{\left[{\text{NaHSO}}_{4}\right]}_{0}+{\left[{\mathrm{RS}}_{2}O_{3}\mathrm{Na}\right]}_{0}}{1+\frac{{\left[{\text{NaHSO}}_{4}\right]}_{0}}{{\left[{\mathrm{RS}}_{2}O_{3}\mathrm{Na}\right]}_{0}}\exp\;(kt({\left[{\text{NaHSO}}_{4}\right]}_{0}+{\left[{\mathrm{RS}}_{2}O_{3}\mathrm{Na}\right]}_{0}))}$$

from the NMR integrations for reactant **1a** and product **2a** in Figure 1 were fit to these equations and are shown in Figure 2B. The average rate constant *k* extracted from the fits to these equations for a 4.67‐mM solution of hexadecyl thiosulfate in THF‐d_8_ at 25°C was 173.4 ± 1.6 M^−1^ min^−1^. The values of *k* from the individual fits were 181.6 ± 1.9 M^−1^ min^−1^ (fit to Equation (2)) and 165.2 ± 2.6 M^−1^ min^−1^ (fit to Equation (3)).

**FIGURE 3:**
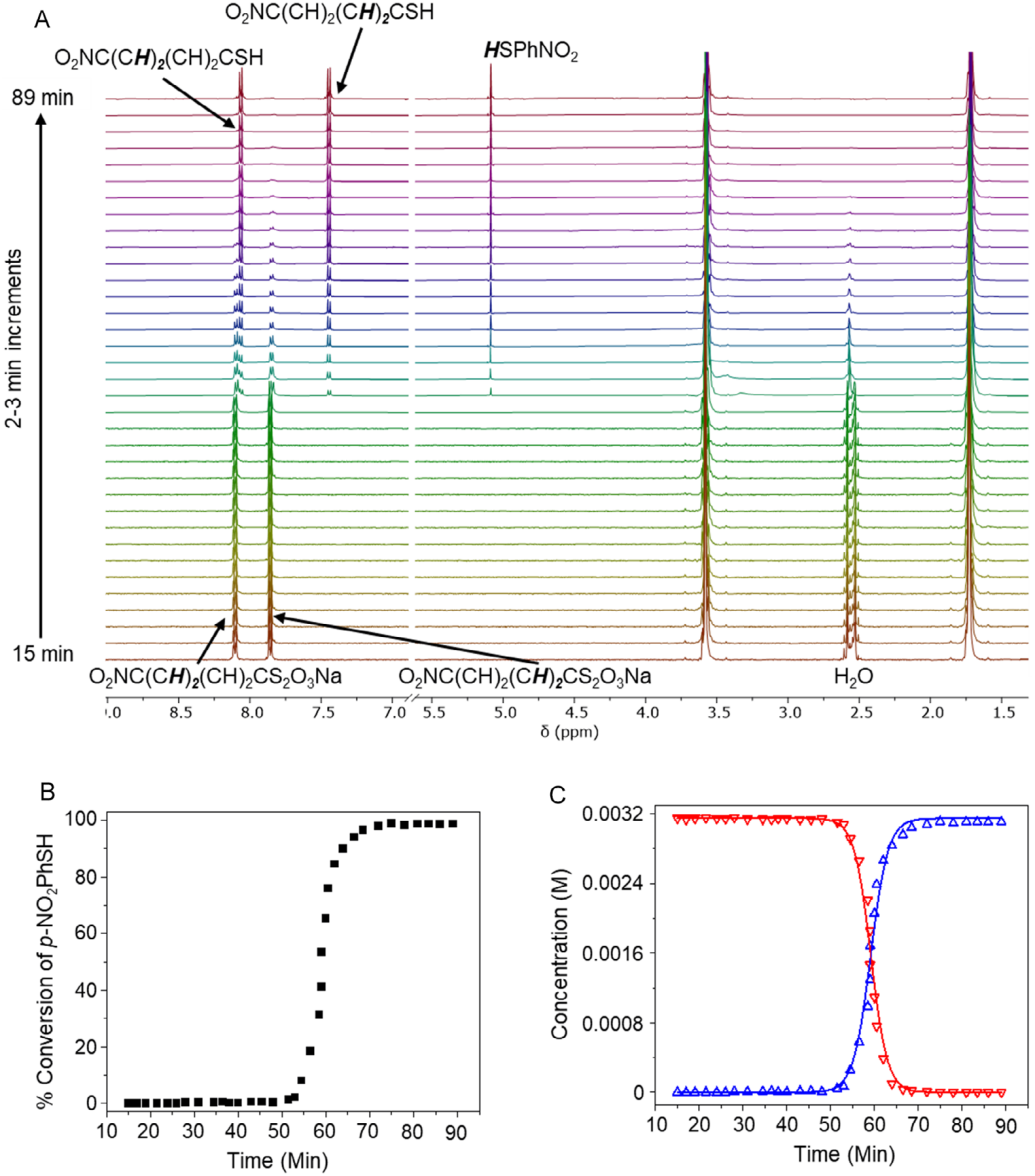
(A) Time‐resolved ^1^H‐NMR scans of 3.15‐mM **1b** in THF‐d_8_ at 2–3‐min intervals. (B) Percent conversion for the hydrolysis of **1b** to produce the corresponding thiol **2b**. (C) Concentration profile of the thiosulfate **1b** (red) and thiol **2b** (blue) during the reaction. The lines are fits to Equations (3) and (2).

The aromatic thiosulfate, p‐nitrophenyl thiosulfate (**1b**), undergoes similar autocatalytic hydrolysis in THF‐d_8_ (≥99.5%), as observed by ^1^H NMR (Figure 3). After the induction period, the resonances due to the 2,2′‐CH protons of the thiosulfate at 7.85 ppm disappeared and were replaced by those of the thiol (**2b**) at 7.45 ppm. The resonances of the 3,3′‐CH protons of the reactant and product differ only very subtly in chemical shift. The emergence of the peak at  ∼5.08 ppm after the induction period corresponds to the thiol proton. As with the alkyl analog, conversion of the thiosulfate to thiol was accompanied by loss of the peak due to water at  ∼2.51 ppm. The 5.5 equivalents of water present in this case were also due to that in the solvent as supplied by the supplier and that crystallized (2.2 equivalents) with the aromatic thiosulfate (Figure S5). Concentrations calculated from the NMR integrations for reactant **1b** and product **2b** in Figure 3A allowed calculation of percent completion of reaction as a function of time (Figure 3B), and fits to the kinetics Equations (3) and (2), respectively (Figure 3C). The average rate constant *k* extracted from the fits to these equations for a 3.15‐mM solution of p‐nitrophenyl thiosulfate in THF‐d_8_ at 25°C was 166.2 ±  4.8 M^−1^ min^−1^. The values of *k* from the individual fits were 167.1 ± 7.6 M^−1^ min^−1^ (fit to Equation (2)) and 165.3 ± 5.7 M^−1^ min^−1^ (fit to Equation (3)).

**FIGURE 4:**
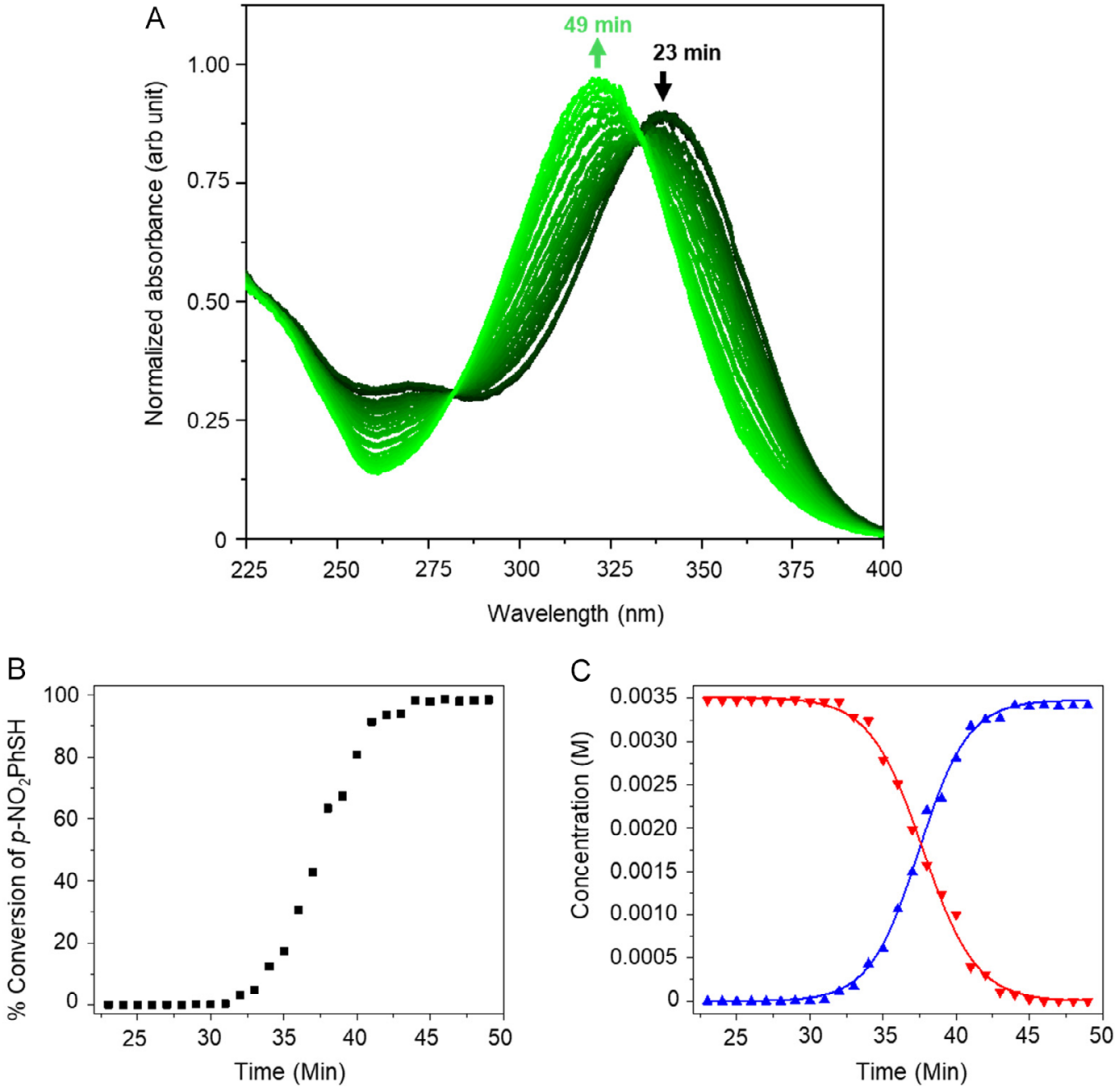
(A) Time‐resolved UV scans of 3.48 mM **1b** in THF at 2–3‐min intervals. (B) Percent conversion for the hydrolysis of **1b** to produce the corresponding thiol **2b**. (C) Concentration profile of the thiosulfate **1b** (red) and thiol **2b** (blue) during the reaction. The lines are fits to Equations (3) and (2).

Absorbances in the near‐UV region also allowed us to measure the kinetics of hydrolysis of p‐nitrophenyl thiosulfate (**1b**) to produce the corresponding thiol (**2b**) using time‐resolved UV spectra. The reaction of a 3.48‐mM solution of **1b** in THF was monitored by collecting spectra at 2–3‐min intervals (Figure 4A). During the course of the reaction, a prominent peak due to the thiosulfate **1b** at 340 nm decreased in intensity and was replaced by one due to the product **2b** at 321 nm. In addition, the absorbance of light at ∼270 nm decreased as the hydrolysis proceeded. Two distinct isosbestic points at 282 and 334 nm were observed, indicating identical molar extinction coefficients for **1b** and **2b** at those wavelengths and consistent with a chromophoric transition from p‐nitrophenylthiosulfate to p‐nitrophenylthiol. Using the absorbances at 340 and 321 nm, Beer–Lambert plots (Figure S6) gave molar absorptivities (ε) of 8.18 × 10^3^ M^−1^ cm^−1^ for **1b** and 1.11 × 10^4^ M^−1^ cm^−1^ for **2b**, which enabled the determination of **1b** and **2b** concentrations as a function of time. As shown in Figure 4B and consistent with the behavior observed for the alkyl analog **1a**, after a ∼32‐min induction period, hydrolysis proceeded rapidly to completion. Fitting the concentration‐time data to Equations (2) and (3) provided an average rate constant, *k*, of 163.2 ± 7.0 M^−1^ min^−1^ (Figure 4C). The individual values of *k* from the two plots were 156.3 ± 10.6 M^−1^ min^−1^ for reactant disappearance and 170.0 ± 9.2 M^−1^ min^−1^ for product appearance.

An alternative explanation for the dramatic rate acceleration observed in THF, relative to water, is the possible formation of reverse micelles [23, 24]. Amphiphilic compounds can encapsulate trace water with their ionic headgroups, while their nonpolar alkyl or aromatic tails orient toward the bulk organic phase. The importance of this behavior in phase‐transfer catalysis [25] and hydrotropic autocatalysis [26] is well documented in the literature. Single‐chained amphiphiles like **1a** described here usually require a cosurfactant (e.g., fatty alcohol) to form inverse micelles, so this explanation may be less likely. Nonetheless, if present (for example, due to the involvement of the product thiol), these dispersed droplets could thus behave like “micro‐reactors” and serve to preconcentrate the reactive species, thereby increasing the rate by increasing the collision frequency significantly above what it would be at the nominal concentrations of the reactants in solution. This type of reactant encapsulation would not be present in water, where normal micelles formed above the critical micelle concentration (CMC) would have the reactant headgroups directed outside the aggregate and in contact with the bulk solution.

**FIGURE 5:**
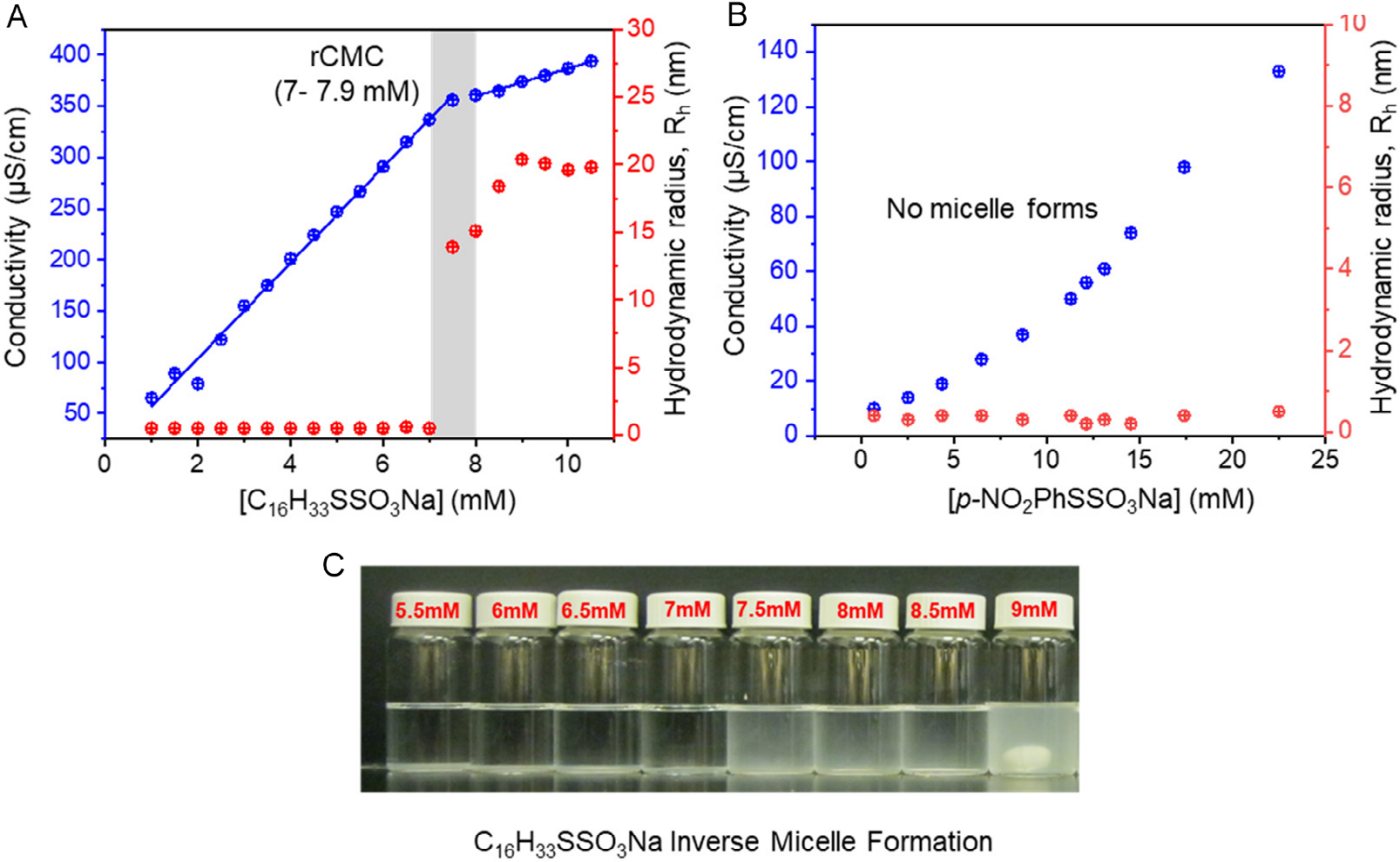
Specific conductivity (blue) and hydrodynamic radius (red) as a function of the concentration of thiosulfate **1a** (A) or **1b** (B) in THF. (C) Opaque appearance of alkyl thiosulfate **1a** at 7.5 mM due to reverse micelle formation.

To test this hypothesis, we attempted to determine the reverse critical micelle concentrations (rCMC) of thiosulfates **1a** and **1b** by measuring the conductivity and hydrodynamic radius of soluble species as a function of concentration in THF. As shown in Figure 5A, the conductivity of **1a** increased linearly up to a discontinuity at 7–8 mM, after which the linear rise had a smaller slope. The rCMC was determined by identifying the intersection of the linear fits to the lines below and above the discontinuity. In contrast, the aromatic thiosulfate **1b**—bearing both ionic S_2_O_3_^−^ and polar NO_2_ substituents but no hydrophobic tail—exhibited a steady rise in conductivity with no discontinuity up to a concentration of 23 mM (Figure 5B), highlighting the absence of reverse micelle formation.

Dynamic light scattering (DLS) measurements are also presented in Figure 5A,B. For alkyl thiosulfate **1a** in THF (≥99.5%), the hydrodynamic radius (*R*_h_) remained near zero at low concentrations, followed by a distinct increase above 7 mM, signaling the onset of reverse micelle formation. This transition was accompanied by a sharp rise in scattering intensity, corresponding to an average reverse micelle radius of approximately 19 nm (Figure 5A). The rCMC inferred from DLS closely matched that estimated from conductivity measurements. In contrast, aromatic thiosulfate **1b** showed no such transition (Figure 5B): the hydrodynamic radius remained negligible across the concentration range up to 23 mM. This lack of aggregation, reinforced by the smooth linear increase in conductivity, points to freely solvated *p‐*O_2_NC_6_H_4_S_2_O_3_^−^ and Na^+^ ions as the dominant species in solution rather than micellar aggregates, which would lead to a discontinuity in conductivity and an increase in scattering intensity. Our conclusions from the conductivity and DLS studies are that around ∼7.5 mM, the long‐chain, aliphatic **1a** reached a concentration sufficient for self‐aggregation, with trace water entrapped within its hydrophilic core to form reverse micelles, and that the aryl **1b** did not. As shown in Figures 2, 3, and 4, both **1a** and **1b** underwent rapid autocatalysis at concentrations well below any rCMC, which effectively rules out reverse micelle formation as responsible for the observed rate accelerations.

For both aliphatic and aromatic thiosulfates, variations in the length of the induction periods were evident, even in controlled experiments. To assess the statistics of these period lengths, we performed high‐throughput screening of thiosulfate **1b** in THF (≥99.5%) using a 96‐well quartz microplate, with each well containing a 100‐μL aliquot from the same stock solution. Based on the kinetics data presented in Figure 4, we used single‐wavelength UV irradiations at 340 nm (*λ*_max_ of **1b**) and 321 nm (*λ*_max_ of **2b**) to monitor the progress of the reaction. An increase in absorbance at 321 nm, accompanied by a decrease in absorbance at 340 nm, indicated the onset of reaction in that particular well.

**FIGURE 6:**
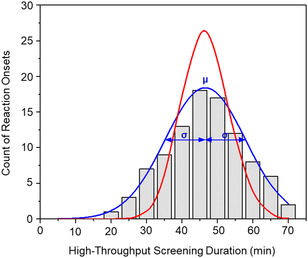
Distribution of hydrolysis induction times for **1b** from high‐throughput screening in a 96‐well microplate fit by a Gaussian model (blue line) and a Poissonian model (red line). Mean reaction time *μ* = 46.6 min with a standard deviation *σ *= ±10.9 min, corresponding to 68% of the observed events according to the Gaussian distribution.

As expected, despite uniform conditions, the induction period varied significantly in length across the wells. Absorption scans taken every 5 min until all 96 reactions were complete revealed a random distribution of induction times across the wells. The experimentally observed distribution of onset events over screening time exhibited a near‐symmetrical shape that closely followed a Gaussian distribution (*χ* ^2^ = 2.02 at 95% confidence band, blue line, Figure 6). A Poisson distribution (*χ* ^2^ = 591.92, red line, Figure 6), which would indicate nonlinear stochasticity, gave a much poorer fit and is shown for comparison [27, 28, 29, 30]. We conclude that the distribution of induction periods likely reflects statistical noise that contributes to the confidence interval (e.g., standard deviation) inherent in physical measurements. Based on the Gaussian model, approximately 68% of reactions initiated between 36.5 and 57.5 min, corresponding to one standard deviation (±σ) around the mean induction time (μ) of 46.6 min. The full width at half maximum (FWHM, 2.35 σ) further suggests that ∼ 95% of reactions occurred within the 34‐to‐59‐min window.

In order to assess the role of solvent more thoroughly, we surveyed the length of the induction times for the hydrolysis reactions in other solvents (Table 1). The induction periods in the hydrolysis of **1a** and **1b** in acetone were significantly longer, 2–3 days (Figures S7 and S8), than in THF. The induction time in acetonitrile was even longer, requiring 5–8 days for thiosulfate **1b** (Figure S9), and no reaction was observed for **1a** after 3 weeks. The long induction times, ranging from 2 days to more than a week, for the onset of autocatalysis rendered these systems unsuitable for further kinetics studies at room temperature. In methanol, ethanol, and dimethyl sulfoxide (DMSO), hydrolysis was not observed, even though the reactant thiosulfates were soluble. Other solvents—ethyl acetate, 1,2‐dimethoxyethane, and chloroform—did not provide sufficient solubility for thiosulfates **1a** and **1b** at room temperature.

**TABLE 1:** Variation in induction period of Bunte salt hydrolysis: solvent effect monitored over an 8‐day period.

Solvent	Induction time	Observation
THF	20–70 min (**1a** and **1b**)	Fast hydrolysis was observed.
Acetone	2–3 days (**1a** and **1b)**	Hydrolysis initiated between 2 and 3 days.
Acetonitrile	No reaction (**1a**) 5–8 days (**1b**)	No hydrolysis was observed for **1a**; hydrolysis initiated between 5 and 8 days for **1b**.
1,2‐Dimethoxyethane	No reaction	Poor solubility for **1a** and **1b**.
Ethyl acetate	No reaction	Poor solubility for **1a** and **1b**.
Chloroform	No reaction	Poor solubility for **1a** and **1b**.
DMSO	No reaction	Both **1a** and **1b** are soluble.
Methanol	No reaction	Both **1a** and **1b** are soluble.
Ethanol	No reaction	Both **1a** and **1b** are soluble.

## 3 Conclusion

The hydrolysis of alkyl and aromatic thiosulfates in THF, containing trace water, is autocatalytic and orders of magnitude faster at room temperature than in water. The reactions are characterized by a significant induction period prior to the onset of autocatalysis and swift completion of the reaction. Fitting the concentration‐time data to autocatalytic models yielded a second‐order rate constant (post‐induction) for *n*‐hexadecyl thiosulfate that is ∼4.1 × 10^10^ times than that calculated at room temperature, based on activation parameters reported for ethyl thiosulfate in water [13, 14]. This accelerated hydrolysis occurs with only trace water present, ∼3 to 5 equivalents, due to the solvent and waters of crystallization of the reactant thiosulfates.

The Kice mechanism for the hydrolysis shown in Scheme 1 involves the transformation of the charged thiosulfate reactant into an uncharged, nonpolar thiol product via a zwitterionic intermediate. Rate‐limiting bond cleavage of the intermediate then occurs through a transition state of diminishing charge and polarity. Previous researchers have attributed rate enhancements to either a more favorable entropy [13] or enthalpy [17, 18, 19, 20, 21, 22] of activation in nonaqueous solvents. These differences may reflect differences in solvation of the reactant; the transition state, which reflects a diminishing polarity that is more compatible with the nonaqueous solvent than with water; or both. The effect of such changes in the compatibility of a solute and its solvation shell on the kinetics of a chemical reaction has potential relevance to important areas ranging from enzyme catalysis to the formation of fossil fuels by conversion of organic matter derived from living organisms into fossil fuels. In the latter example, the earliest stages of this process involve dehydration of hydrophilic compounds, such as carbohydrates and other biomolecules, to produce increasingly hydrophobic polymeric materials [25, 31, 32].

Although micellization has been linked to autocatalysis, phase‐transfer catalysis, and enhancing hydrolysis of amphiphiles, it had no discernible impact in the systems studied. Hydrolysis of **1a** occurred well below its reverse critical micelle concentration (rCMC), as determined by conductivity and dynamic light scattering, and **1b** showed no formation of reverse micelles at all over the concentration range measured. The induction period prior to the onset of reaction of **1a** and **1b** in THF varied significantly, from minutes to hours for controlled reactions. We examined this feature of the reactions using high‐throughput screening of identical reactions to monitor the induction times across 96‐microplate wells. The probability distribution of 96 induction periods followed a Gaussian distribution peaking at 46.6 min, with 68% of reactions initiating between 36.5 and 57.5 min. A survey of other solvents found a range of behavior, all with slower kinetics. Hydrolysis was not observed in the polar protic solvents EtOH and MeOH, and highly polar aprotic DMSO. In acetone and acetonitrile, the thiosulfates displayed moderate reactivity; however, the onset of hydrolysis in these solvents was very slow, typically requiring 3–8 days.

## Supporting Information

Additional supporting information can be found online in the Supporting Information Section. **Supporting Fig. S1:** ^1^H NMR spectra of C_16_H_33_S_2_O_3_Na, **1a**, in methanol‐d_4_. **Supporting Fig. S2:** ^1^H NMR spectra of p‐O_2_NC_6_H_4_S_2_O_3_Na, **1b**, in methanol‐d_4_. **Supporting Fig. S3:** Thermogravimetric analysis showing weight (%) and derivative weight (%/°C) as a function of temperature. A. Weight loss of 0.59 mg between 60°C–100°C corresponds to 3.74% water content in a 15.67 mg sample of C_16_H_33_S_2_O_3_Na, B. Weight loss of 1.52 mg between 115°C–150°C corresponds to 13.4% water content in an 11.32 mg sample of p‐O_2_NC_6_H_4_S_2_O_3_Na. **Supporting Fig. S4:** ^1^H NMR spectrum of 4.7‐mM C_16_H_33_S_2_O_3_Na (**1a**) in THF‐d_8_ with a **1a**‐to‐water integration ratio of 1: 3.9. **Supporting Fig. S5:** ^1^H NMR spectrum of 3.2‐mM p‐O_2_NC_6_H_4_SSO_3_Na (**1b**) in THF‐d_8_ with a **1b**‐to‐water integration ratio of 1: 5.5. **Supporting Fig. S6:** Beer‐Lambert plots of absorbance as a function of concentration in THF for: **A**. p‐O_2_NC_6_H_4_S_2_O_3_Na (λ_max_ 340 nm); and **B**. p‐O_2_NC_6_H_4_SH (λ_max_ 321 nm). **Supporting Fig. S7:** Time‐resolved ¹H NMR spectra during hydrolysis of **1a** in acetone‐d_6_. The resonance due to the α‐CH_2_ protons of **1a** at 2.92 was eventually replaced by that due to the corresponding protons in **2a** at 2.61 ppm; likewise, the resonance for β‐CH_2_ protons of **1a** at 1.71 was replaced by those of **2a** at 1.58 ppm; and the water signal at ∼2.8 ppm disappeared upon completion of the hydrolysis. **Supporting Fig. S8:** Time‐resolved ¹H NMR spectra during hydrolysis of **1b**, in acetone‐d_6_. Disappearance of the 2,2’ resonances of **1b** at 7.8 ppm, appearance of the corresponding **2b** resonance at 7.5 ppm, and loss of the water signal at ∼2.8 ppm confirmed the complete hydrolysis to thiol product within 2 days. **Supporting Fig. S9:** Hydrolysis of **1b** in acetonitrile. Upper figure: Time‐resolved ¹H NMR spectra of **1b** in acetonitrile‐d_6_ exhibited the disappearance of the 2,2’ resonances of **1b** at 7.86 ppm, appearance of the corresponding **2b** resonance at 7.47 ppm, and loss of the water signal at ∼2.23 ppm after 5 days. Lower Figure: Time‐resolved UV spectra of **1b** in acetonitrile showed λ_max_ of **1b** at 337 nm was replaced by that of **2b** at 319 nm after more than a week. **Supporting Table S1** **:** Calculated water of crystallization in compounds **1a** and **1b** based on TGA analysis. **Supporting Table S2** **:** Conductivity and hydrodynamic radius of C_16_H_33_S_2_O_3_Na (**1a**) in THF. **Supporting Table S3** **:** Conductivity and hydrodynamic radius of p‐O_2_NC_6_H_4_S_2_O_3_Na (**1b**) in THF. **Supporting Table S4** **:** Initiation of hydrolysis across N wells as a function of screening time.

## Conflicts of Interest

The authors declare no conflicts of interest.

## Supporting information

Supplementary Material

## Data Availability

The data that support the findings of this study are available in the supplementary material of this article.
