# Supplementary material for: Large Solvent‐Dependent Rate Enhancement of Autocatalytic Hydrolysis of Alkyl and Aryl Thiosulfates
Source: ChemistryOpen. 2026 Apr 9;15(4):e202500552. doi: 10.1002/open.202500552 (PMC13064793; doi:10.1002/open.202500552)
Supplement: Supplementary file 1 — Supplementary Material [file OPEN-15-e202500552-s001.pdf]

Supplementary Information for:

Large Solvent-Dependent Rate Enhancement of Autocatalytic Hydrolysis of Alkyl and Aryl Thiosulfates

Fatema Binte Amin,[a] Katherine M. Kressler,[a] Gregory S. Ferguson[a],[b]*

[a] Department of Chemistry, Lehigh University, Bethlehem, PA 18015, USA
[b] Department of Materials Science & Engineering, Lehigh University, Bethlehem, PA 18015, USA
E-mail: gf03@lehigh.edu

Table of Contents

Materials and Synthesis 2
Thermogravimetric Analysis (TGA) 4
Residual Water in THF-d_8 5
Kinetics Experiments (^{1}H NMR and UV absorption) 6
Reverse Critical Micelle Concentration (rCMC) Determination (Conductivity and Dynamic Light Scattering) 7
High-Throughput Screening of Induction Times 10
Studies of Hydrolysis in Other Solvents 11
References 14

Materials and Synthesis.

General. Tetrahydrofuran (Mallinckrodt, 99%), ethyl acetate (≥99.5%, Sigma-Aldrich), and acetonitrile (>99.5%, Sigma-Aldrich) were dried using a PureSolv system (Innovative Technology, Inc.). Acetone (99%, Carolina Biological), Chloroform (≥99%, Sigma-Aldrich), 1,2-dimethoxyethane (≥99.5%, Sigma-Aldrich), ethanol (200 Proof, lab Alley), and methanol (>99.6%, Sigma-Aldrich) were dried over activated 4 Å molecular sieves (Alfa Aesar). 1-Bromohexadecane (98%) and sodium thiosulfate pentahydrate (99%) were used as received from Alfa Aesar, and sodium metabisulfite (97%) and 1-nitro-4-[(4-nitrophenyl)dithio]benzene (97%) were used as received from Oakwood Chemicals. Ultrapure water (18 MΩ·cm resistivity) was obtained using a Purelab Prima system (Elga). Deuterated solvents, THF-d_8 (≥99.5%), acetone-d_6 (99.9%), methanol-d_4 (99.5%), ethanol-d_6 (99%), and acetonitrile-d_3 (>99.8%), were supplied from Cambridge Isotope Laboratories and used as received.

Synthesis of *n*-hexadecyl thiosulfate (1a): Compound **1a** was synthesized and characterized according to previously reported procedures.[1] Thermogravimetric analysis (TGA) and ^{1}H NMR confirmed that the product was approximately a monohydrate. ^{1}H NMR (400 MHz, CD_3OD): $C\boldsymbol{H_3}(CH_2)_{15}SSO_3Na$, 0.88-0.91 (t, 3H); $CH_3(C\boldsymbol{H_2})_{13}(CH_2)_2SSO_3Na$, 1.29 (m, 26H); $CH_3(CH_2)_{13}C\boldsymbol{H_2}CH_2SSO_3Na$, 1.72-1.78 (m, 2H); $CH_3(CH_2)_{13}CH_2\boldsymbol{CH_2}SSO_3Na$, 3.04-3.07 (t, 2H).

Synthesis of sodium 4-nitrophenyl thiosulfate (1b). Compound **1b** was synthesized following the method reported by Lecher and Hardy.[2] Sodium metabisulfite (1.25 g, 6.57 mmol) was dissolved in 1 mL of water and added to a solution of 4-nitrophenyl disulfide (0.92 g, 3.0 mmol) in 15 mL of methanol. The mixture was refluxed for approximately 8 h until homogeneous, then cooled to room temperature. Sodium hydroxide pellets (0.12 g, 3.0 mmol) were added to neutralize excess sodium bisulfite. The resulting slurry was filtered, and the solid was washed with methanol. The methanol from the filtrate was removed by rotary evaporation, and the residue was redissolved in dry methanol. After filtration to remove insoluble sulfite, the pale-yellow crystalline product was isolated by rotary evaporation and washed twice with anhydrous methanol. The solid was dried and stored under refrigeration. The isolated yield was 85%. Thermogravimetric analysis (TGA) and ^{1}H NMR confirmed that the product was approximately a dihydrate. ^{1}H NMR (400 MHz, CD_3OD): *p*-$O_2NC(C\boldsymbol{H})_2(CH)_2CSSO_3Na$, 8.20 (m, 2H), *p*-$O_2NC(CH)_2(C\boldsymbol{H})_2CSSO_3Na$, 7.89 (m, 2H).

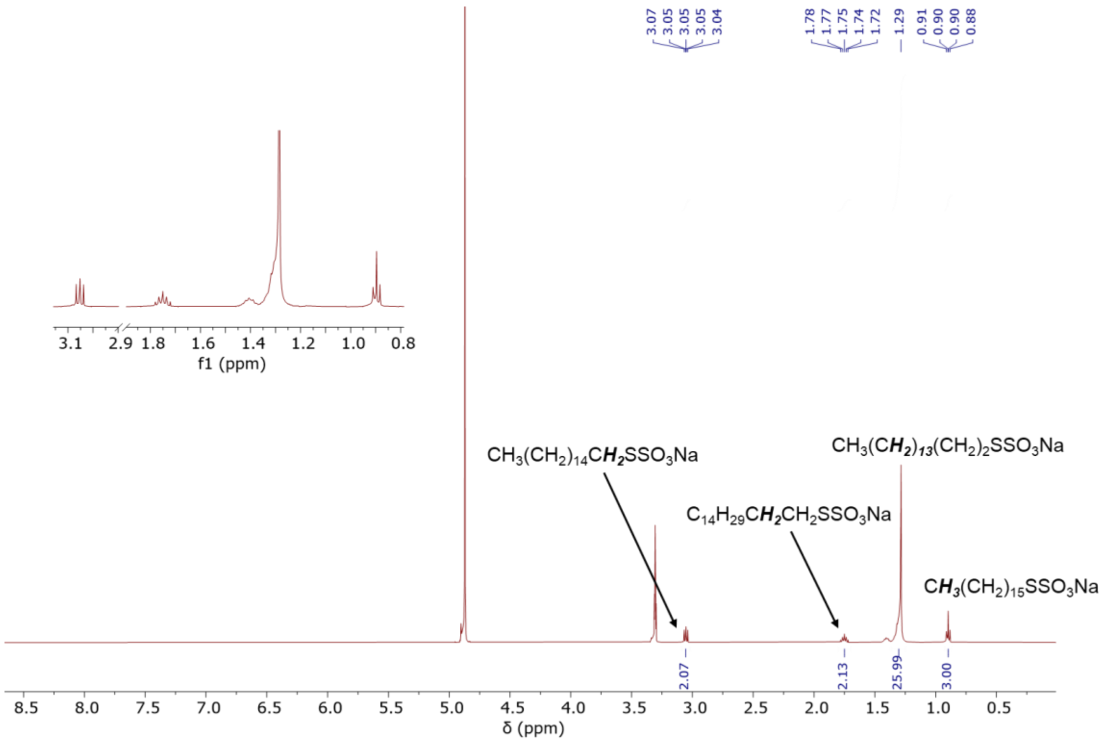

Figure S1. ^{1}H NMR spectra of $C_{16}H_{33}S_2O_3Na$, **1a**, in methanol-d$_4$.

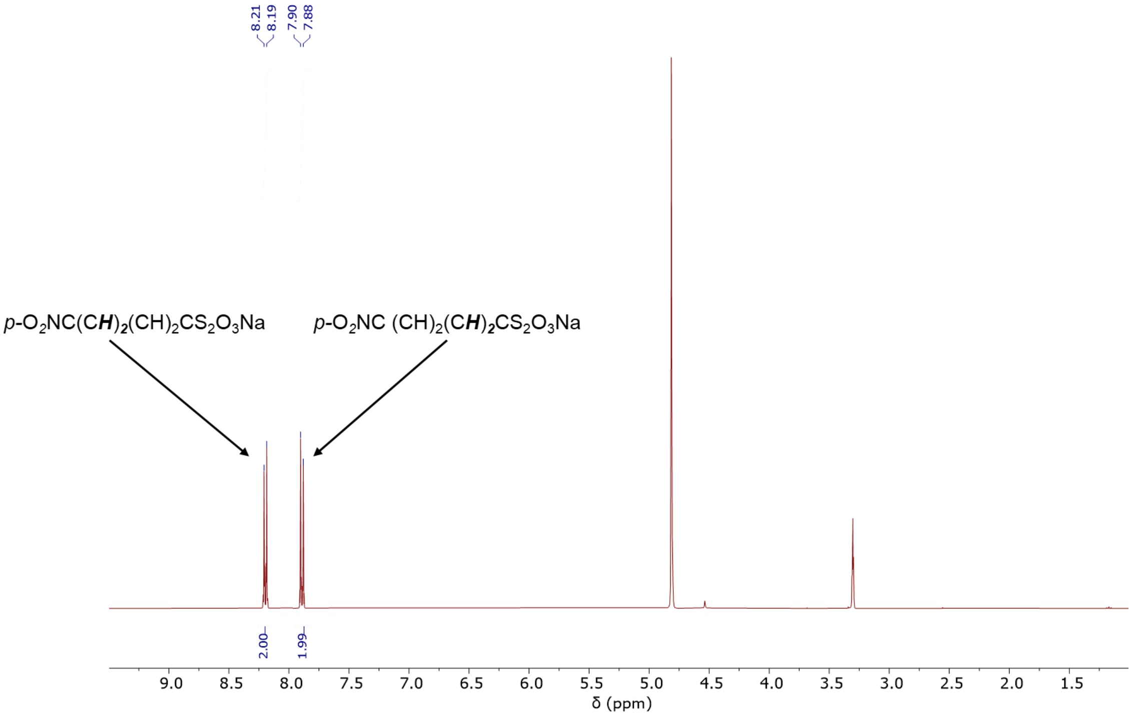

Figure S2. ^{1}H NMR spectra of *p*-$O_2NC_6H_4S_2O_3Na$, **1b**, in methanol-d$_4$.

Thermogravimetric Analysis (TGA).

Thermogravimetric analysis (TGA) was conducted using a TA Instruments Q500 analyzer to determine the crystalline water content of recrystallized Bunte salts. The instrument was calibrated with copper(II) acetate monohydrate [$Cu(OAc)_2 \cdot H_2O$] and operated under a nitrogen atmosphere to prevent thermal decomposition, which is observed for Bunte salts at elevated temperatures in air. Samples were heated at a rate of 10 °C/ min up to 250 °C. A mass loss of 3.74% (corresponding to 0.79 equivalents of water) was observed for $C_{16}H_{33}S_2O_3Na$, and a loss of 13.4% (2.2 equivalents of water) was measured for *p*-$O_2NC_6H_4S_2O_3Na$.

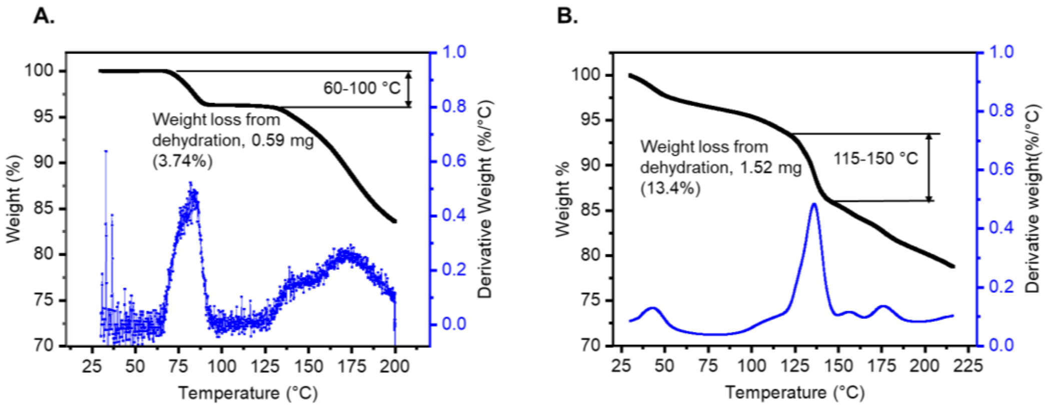

Figure S3. Thermogravimetric analysis showing weight (%) and derivative weight (% /°C) as a function of temperature. A. Weight loss of 0.59 mg between 60–100 °C corresponds to 3.74% water content in a 15.67 mg sample of $C_{16}H_{33}S_2O_3Na$, B. Weight loss of 1.52 mg between 115–150 °C corresponds to 13.4% water content in an 11.32 mg sample of *p*-$O_2NC_6H_4S_2O_3Na$.

Table S1. Calculated water of crystallization in compounds **1a** and **1b** based on TGA analysis.

**R**S_2O_3Na	Anhydrous MW	Sample mass (g)	Water loss (g)	Moles of RS_2O_3Na = $\frac{\text{(Sample mass} - \text{Water loss)}}{\text{Anhydrous MW}}$	Moles of H_2O	H_2O **:** RS_2O_3Na
$C_{16}H_{33}$	360.55	0.015670	0.000585	0.000042	0.000033	0.79
p-$O_2NC_6H_4$	257.22	0.011320	0.001520	0.000038	0.000084	2.2

Residual Water in THF-d_8.

The ^{1}H NMR spectrum of $C_{16}H_{33}S_2O_3Na$ (**1a**) in THF-d_8 contained a narrow symmetrical singlet due to residual H_2O. A 4.7-mM solution revealed a ratio of 1 **:** 3.9 (**1a : **H_2O), as shown in Figure S4. Accounting for the 0.79 equivalents of crystallized water (hydrates) per equivalent of $C_{16}H_{33}SSO_3Na$ revealed by thermogravimetry, the remaining ~3.1 equivalents of water originate from the solvent.

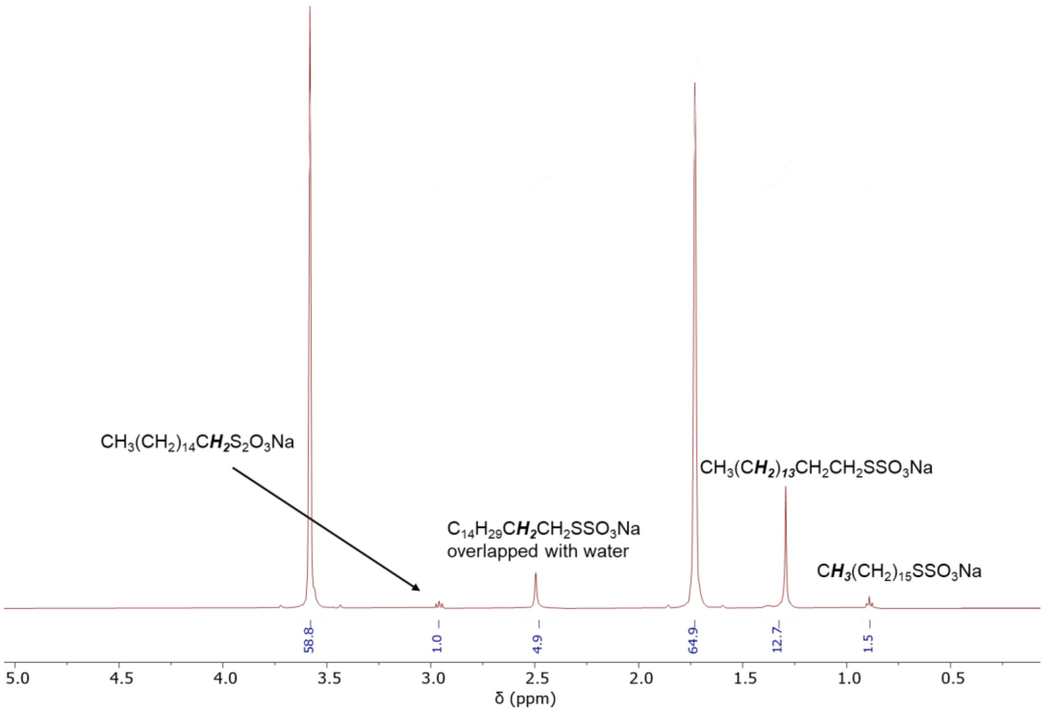

Figure S4. ^{1}H NMR spectrum of 4.7-mM $C_{16}H_{33}S_2O_3Na$ (**1a**) in THF-d_8 with a **1a**-to-water integration ratio of 1 : 3.9.

The ^{1}H NMR spectrum of a 3.2-mM solution of *p*-$O_2NC_6H_4S_2O_3Na$ (**1b**) in THF-d_8 revealed a ratio of 1 **:** 5.5 (**1b :** H_2O), as shown in Figure S5. Considering the 2.2 equivalents of crystallized water (hydrates) per equivalent of *p*-$O_2NC_6H_4S_2O_3Na$, the remaining ~3.3 equivalents of water originate from the THF-d_8 solvent.

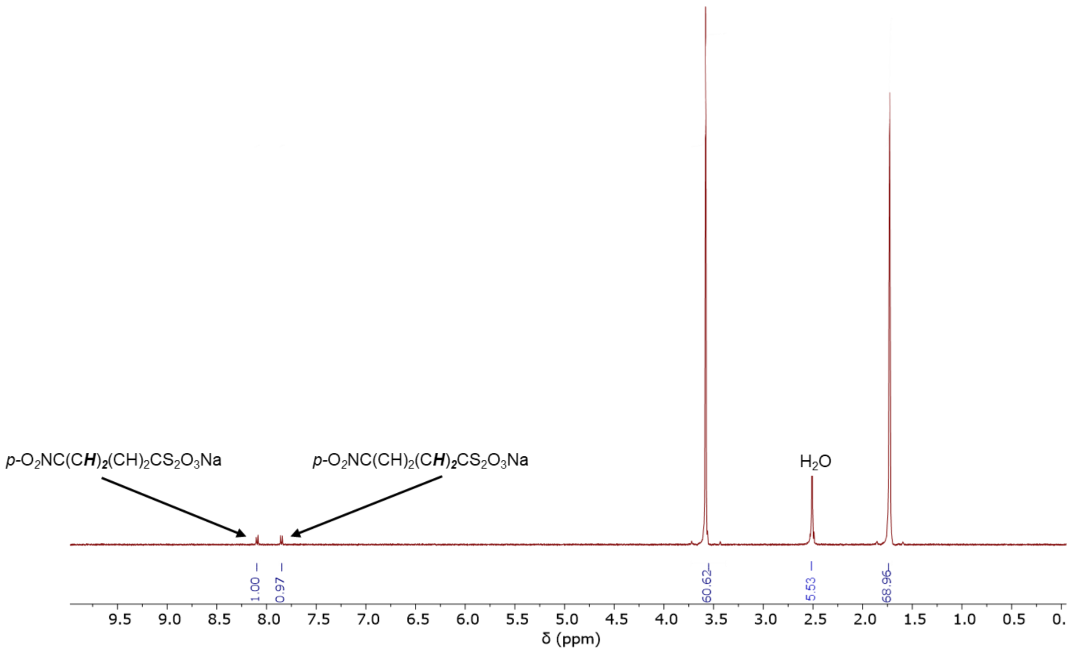

Figure S5. ^{1}H NMR spectrum of 3.2-mM *p*-$O_2NC_6H_4SSO_3Na$ (**1b**) in THF-d_8 with a **1b**-to-water integration ratio of 1 : 5.5.

Kinetics Experiments.

^{1}H NMR. The hydrolysis of $C_{16}H_{33}S_2O_3Na$ (**1a**) in THF-d_8 was monitored by time-resolved ^{1}H NMR spectroscopy (Bruker, 400 MHz) at 1-2 minute intervals. A temperature control unit maintained the reaction at 25 °C. The progress of hydrolysis was tracked by the disappearance of the *α*-CH_2 proton resonance at 2.96 ppm for **1a** and the appearance of the corresponding thiol (**2a**) resonance at 2.47 ppm, as shown in Figure 1 of the main article. We infer, from a 1-proton increase in integration, that the resonance due to the thiol proton (S*H*) in the product **1b** overlaps that of the *β*-CH_2 protons just below 1.60 ppm.

Similar time-resolved ^{1}H NMR scans of **1b** at 2-3 minute intervals during its hydrolysis in THF-d_8 showed initial 2,2'-CH resonances at 7.85 (m, 2H); upon complete hydrolysis to the thiol **2b**, the corresponding resonance appeared at 7.45 ppm. The small difference in chemical shift between the 3,3'-CH protons of the reactant and product prevented their use in monitoring the kinetics of the reaction. (Figure 3A of the main article).

The decrease in the intensity and eventual disappearance of the water signal at ~2.5 ppm in both cases reflects the hydrolysis and concomitant acidification of the solution.

UV Absorption. The kinetics of hydrolysis of **1b** in THF was also monitored by UV absorption spectroscopy (Cary 5000 UV-Vis-NIR spectrophotometer), with scans recorded every 2–3 minutes in 0.1-cm pathlength (*l*) cuvettes. An initial scan (at ~23 min) showed a strong absorbance at 340 nm for **1b**. As the reaction progressed, the emergence of a characteristic band at 321 nm indicated the formation of the thiol product **2b**, as detailed in the main article.

Absorbances at 340 nm (λ_{max}) for **1b** and 321 nm (λ_{max}) for **2b** were plotted as a function of concentration in THF to determine molar absorptivities (ε) of 8.18×10^3 $M^{-1}cm^{-1}$ and 1.11×10^4 $M^{-1}cm^{-1}$, respectively, according to Beer–Lambert law ($C = A/\varepsilon l$). These values were used to calculate the concentrations of **1b** and **2b** at specific times in the kinetics profiles shown in Figures 4B and 4C of the main article.

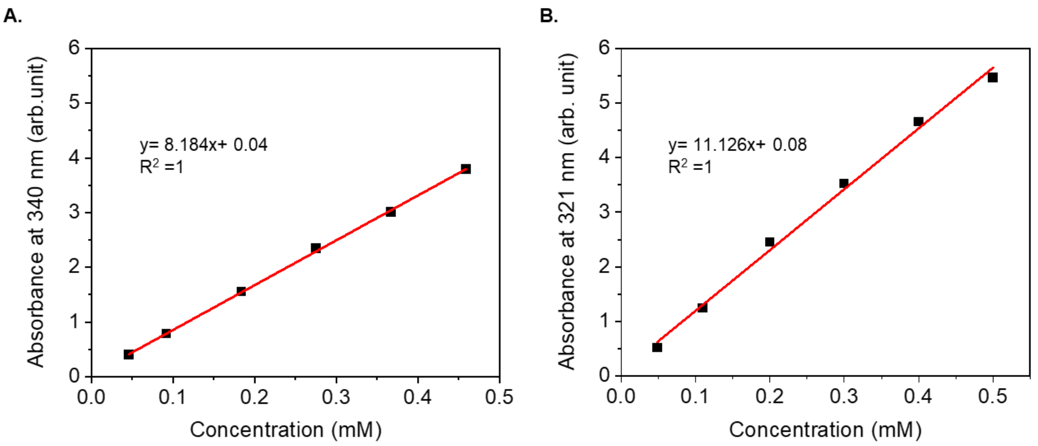

Figure S6. Beer-Lambert plots of absorbance as a function of concentration in THF for: **A**. *p*-$O_2NC_6H_4S_2O_3Na$ (λ_{max} 340 nm); and **B**. *p*-$O_2NC_6H_4SH$ (λ_{max} 321 nm).

Reverse Critical Micelle Concentration (rCMC) Determination.

Conductivity Measurements. The conductivity of THF solutions of **1a** and **1b** was measured using an Oakton Instruments 35604-10 EC 100 conductivity meter (range: 1–200,000 µS/cm) as a function of concentration. The probe was first calibrated with a standard KCl reference solution (0.001 M, 147 µS/cm). Between trials, it was rinsed with distilled water and gently dried with a Kimwipe®.

Dynamic Light Scattering. An ALV/CG3 Dynamic Light Scatterer (DLS) was used to measure the scattered intensity at 90° and determine the intensity-weighted hydrodynamic radius (R_h) of **1a** and **1b** in THF as a function of concentration. Each concentration was measured in triplicate, and the averaged R_h values are reported in Tables S2 and S3.

Table S2. Conductivity and hydrodynamic radius of $C_{16}H_{33}S_2O_3Na$ (**1a**) in THF.

Concentration (mM)	Conductivity (μS/cm)	Hydrodynamic Radius (nm)
1	65	0.5
1.5	89	0.5
2	79	0.5
2.5	122	0.5
3	155	0.5
3.5	175	0.5
4	201	0.5
4.5	224	0.5
5	247	0.5
5.5	267	0.5
6	291	0.5
6.5	315	0.6
7	337	0.5
7.5	356	13.9
8	361	15.1
8.5	365	18.4
9	374	20.4
9.5	380	20.1
10	387	19.1
10.5	394	19.9

Table **S3.** Conductivity and hydrodynamic radius of *p*-$O_2NC_6H_4S_2O_3Na$ (**1b**) in THF.

Concentration (mM)	Conductivity (μS/cm)	Hydrodynamic Radius (nm)
0.7	10	0.4
2.54	14	0.3
4.36	19	0.4
6.5	28	0.4
8.7	37	0.3
11.3	50	0.4
12.13	56	0.2
13.1	61	0.3
14.53	74	0.2
17.4	98	0.4
22.5	133	0.5

High-Throughput Screening of Induction Times.

High-throughput screening of the onset of hydrolysis of *p*-$O_2NC_6H_4S_2O_3Na$ in THF was conducted using a Tecan Infinite 200 PRO multiplate reader. A stock solution of 0.4 mM **1b** was pipetted into 96 wells of a quartz microplate (supplied by Analytical Sales and Services), with each well receiving a 100-μL aliquot. Absorbance measurements were performed at 340 and 321 nm at 5-minute intervals until the hydrolysis was complete in all wells (Table S4). No stirring was applied, and the temperature was maintained at 25 °C. The distribution of induction periods was fitted by Gaussian and Poisson models in OriginPro 2025 Graphing and Analysis Software.

Table S4. Initiation of hydrolysis across *N* wells as a function of screening time.

Time (min)	**Count of wells showing hydrolysis onset** since the preceding measurement, *N*
20	1
25	3
30	7
35	9
40	13
45	18
50	17
55	12
60	8
65	6
70	2

Studies of Hydrolysis in Other Solvents.

For both $C_{16}H_{33}S_2O_3Na$ (**1a**, Figure S7) and *p*-$O_2NC_6H_4S_2O_3Na$ (**1b**, Figure S8), hydrolysis in acetone-d_6 initiated after 2-3 days.

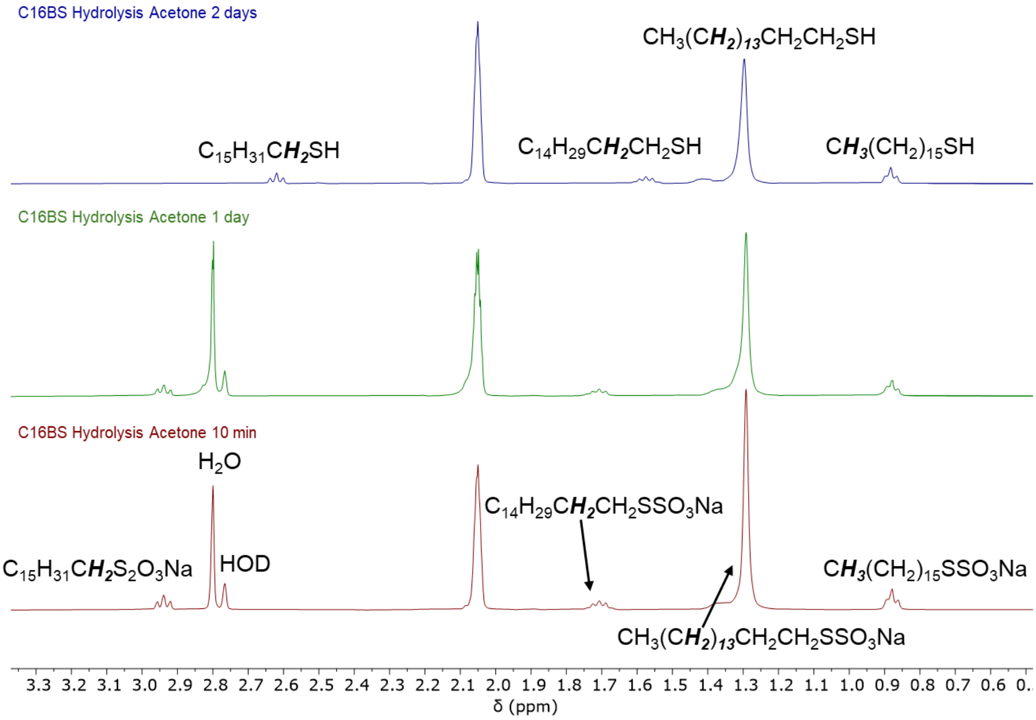

Figure S7. Time-resolved ¹H NMR spectra during hydrolysis of **1a** in acetone-d_6. The resonance due to the *α*-CH_2 protons of **1a** at 2.92 was eventually replaced by that due to the corresponding protons in **2a** at 2.61 ppm; likewise, the resonance for *β*-CH_2 protons of **1a** at 1.71 was replaced by those of **2a** at 1.58 ppm; and the water signal at ~2.8 ppm disappeared upon completion of the hydrolysis.

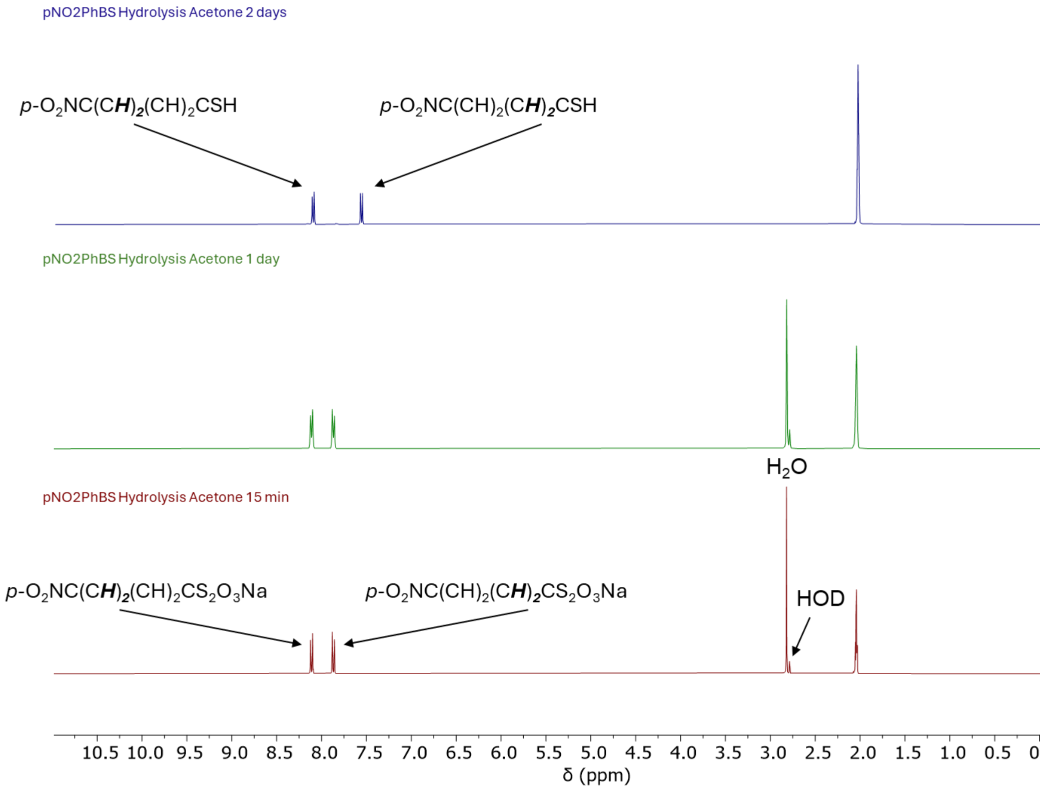

Figure S8. Time-resolved ^{1}H NMR spectra during hydrolysis of **1b**, in acetone-d_6. Disappearance of the 2,2' resonances of **1b** at 7.8 ppm, appearance of the corresponding **2b** resonance at 7.5 ppm, and loss of the water signal at ~2.8 ppm confirmed the complete hydrolysis to thiol product within 2 days.

Although the ^{1}H NMR spectra of **1a** remained unchanged in acetonitrile-d3 for weeks, time-resolved ^{1}H NMR and UV scans revealed eventual, complete hydrolysis of **2a** to **2b** in acetonitrile after between 5 and 8 days (Figure S9).

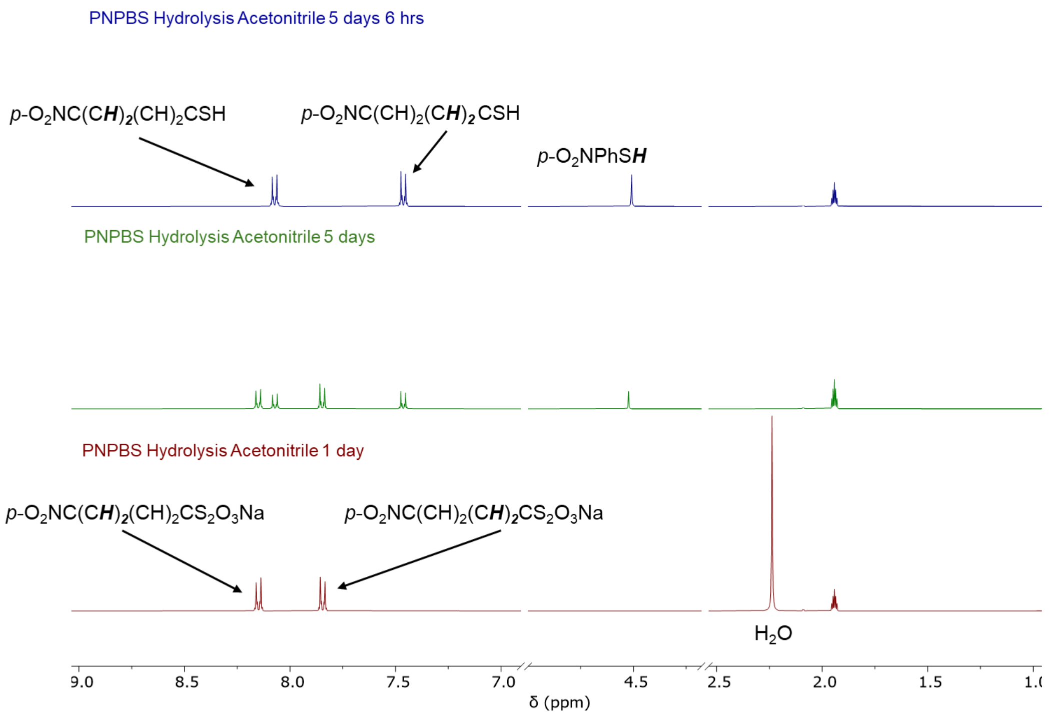

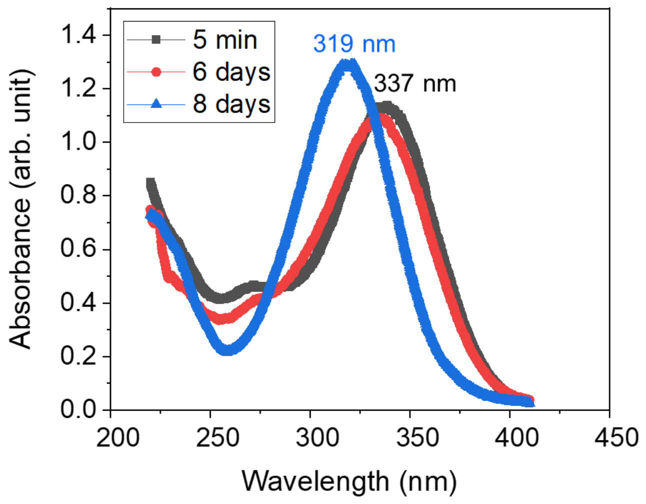

Figure S9. Hydrolysis of **1b** in acetonitrile. Upper figure: Time-resolved ^{1}H NMR spectra of **1b** in acetonitrile-d_6 exhibited the disappearance of the 2,2' resonances of **1b** at 7.86 ppm, appearance of the corresponding **2b** resonance at 7.47 ppm, and loss of the water signal at ~2.23 ppm after 5 days. Lower Figure: Time-resolved UV spectra of **1b** in acetonitrile showed λ_{max} of **1b** at 337 nm was replaced by that of **2b** at 319 nm after more than a week.

References.

[1] J. P. Labukas, T. J. H. Drake, G. S. Ferguson, *Langmuir* **2010**, *26*, 9497–9505.

[2] H. Z. Lecher, E. M. Hardy, *J. Org. Chem.* **1955**, *20*, 475–487.
